# Effect of co-administration of BRL-37344 and tadalafil on reduction of overactive bladder symptoms after induction of detrusor overactivity in mice^1^

**DOI:** 10.1590/s0102-8650201900205

**Published:** 2019-02-28

**Authors:** Marcos Fiuza de Carvalho, Thuliermes Lopes Pamplona, Márcio Alencar Barreira, Francisco Vagnaldo Jacuru Fechine, Lúcio Flávio Gonzaga-Silva, Ricardo Reges Maia de Oliveira

**Affiliations:** I Fellow Master degree, Postgraduate Program in Medical Surgical Sciences, Universidade Federal do Ceará (UFC), Fortaleza-CE, Brazil. Conception and design of the study; technical procedures; acquisition, interpretation and analysis of data; manuscript preparation and writing.; II PhD, UFC. Fortaleza-CE, Brazil. Acquisition of data, technical procedures.; III Fellow PhD degree, Postgraduate Program in Medical Surgical Sciences, UFC, Fortaleza-CE, Brazil. Manuscript preparation and writing, critical revision.; IV PhD, Researcher, Nucleus of Research and Development of Medicines, Department of Pharmacology, UFC, Fortaleza-CE, Brazil. Analysis and interpretation of data, statistical analysis.; V PhD, Urologist, Department of Surgery, UFC, Fortaleza-CE, Brazil. Conception and design of the study, interpretation and analysis of data, critical revision, final approval.

**Keywords:** Urinary Bladder, Overactive, Nitric Oxide, Muscle Relaxation, Mice

## Abstract

**Purpose:**

To evaluate the impact of the combination of BRL 37344 and tadalafil (TDF) on the reduction of overactive bladder (OB) symptoms.

**Methods:**

Thirty mice were randomized into 5 groups (G) of 6 animals each. L-NAME was used to induce DO. G1: Control; G2: L-NAME; G3: L-NAME + TDF; G4: L-NAME + BRL 37344; G5: L-NAME + TDF + BRL 37344. After 30 days of treatment, the animals were submitted to cystometry to evaluate non-voiding contractions (NVC), threshold pressure (TP), baseline pressure (BP), frequency of micturition (FM) and threshold volume (TV). Differences between the groups were analyzed with ANOVA followed by the Tukey test.

**Results:**

NVC increased in G2 (4.33±2.58) in relation to G1 (1.50±0.55). NVC decreased in G3 (2.00±1.10), G4 (1.50±1.52) and G5 (2.00±1.26) compared to G2 (p<0.05). FM decreased in G3 (0.97±0.71), G4 (0.92±0.38) and G5 (1.05±0.44) compared to G2 (p<0.05). However, the combination of TDF and BRL37344 was not more effective at increasing NVC and improving FM than either drug alone. The five groups did not differ significantly with regard to TV.

**Conclusion:**

The combination of BRL 37344 and TDF produced no measurable additive effect on reduction of OB symptoms.

## Introduction 

 Overactive bladder (OB) is a highly prevalent symptom condition that affects millions of US men and women. Costs for the management of OB continue to rise and represents a significant public health burden to the USA^1^. A Brazilian study showed a high prevalence of OB (18.9%), leading to impaired quality of life and sexual function^2^. The gold standard treatment of this pathology is the use of medications with anti-muscarinic action^3^. However, some patients discontinue treatment because they do not present a good response and do not tolerate adverse effects (dry mouth, constipation, blurred vision)^4^. The limitations of anti-muscarinic therapy indicate the need for effective and well tolerated options in the treatment of detrusor overactivity (DO).

 β3 adrenergic receptor agonists (β3-AR) have emerged as a promising class of drugs by relaxing the detrusor smooth muscle (DSM) of humans^5^ and animals^6^ ^,^ ^7^. BRL 37344 is a phenylethononamine of the first generation β3 adrenergic family^8^. Another option in the treatment of DO is Tadafila, which is a selective inhibitor of phosphodiesterase type 5 (PDE-5) that potentiates the action of nitric oxide (NO) at a concentration of 4mg/kg^9^. 

 Nω-nitro-L-arginine methyl ester hydrochloride (L-NAME) at a dose of 60 mg / kg / day leads to DO by inhibiting NO in the animal bladder^10^. The NO leads to a dissociation of actin and myosin fibers by reduction of intracellular calcium and, consequently, causes relaxation of the bladder smooth muscle. The NO also stimulates the activity of phosphodiesterases that metabolize adenosine cyclic monophosphate (cAMP) and guanosine cyclic monophosphate (cGMP)^11^. CAMP and cGMP are involved in the relaxation of smooth muscle through the control of ion channels and phosphorylation of certain proteins^12^. 

 β3-AR agonists stimulate the production of cAMP^13^, whereas PDE inhibitors prevent cAMP and cGMP degradation^14^. There is evidence that PDE-5 is by far the most important PDE in cGMP signaling^12^. Thus, the association of the two drugs could have a synergism in DSM relaxation.

 To our knowledge, no other study has evaluated the effect of co-administration of PDE-5 inhibitors and β3-AR agonists in models of DO in vivo. The objective of this in vivo experimental study was therefore to evaluate the impact of the combination of BRL 37344 (a β3-AR agonist) and tadalafil (a PDE_5_ inhibitor) on the reduction of OB symptoms after induction of DO.

## Methods

 The experimental study was performed at the Laboratory of Experimental Surgery, Universidade Federal do Ceará (UFC) after approval by the Ethics and Animal Research Committee (protocol 05/14).

 The sample consisted of 30 male Mus Musculus mice weighing between 40 and 50g. The mice came from the Bioterio of UFC, and were distributed randomly in 5 groups of 6 animals. The mice were kept in polypropylene cages with galvanized zinc-plated wire cover, coated with excelsiors. They were housed in adequate conditions of temperature (average of 25ºC), ventilation, lighting, relative air humidity around 50% and the light and darkness alternating every 12 hours. They received water and *ad libitum* feed.

 Group 1 did not receive any of the medications and served as a control group. 2-5 groups received 60 mg / kg / day L-NAME (Sigma-Aldrich, St. Louis, Missouri, USA) diluted in drinking water^10^. Groups 3 and 4 received 4mg / kg / day of Tadalafila (Cayman, Ann Arbor, Michigan, USA) by oral gavage^9^. In groups 4 and 5, 5mg / kg / intraperitoneal BRL-37344 (Sigma-Aldrich, St. Louis, Missouri, USA) was administered once every 30 minutes prior to cystometry^15^. After 30 days of follow-up, all mice were referred for cystometry**.**

###  Cystometry 

 For cystometry, the animals were anesthetized with Urethane (1.2g/kg) and the carotid artery cannulated for mean arterial blood pressure monitoring. A 1cm incision was made along the midline of the rat abdomen. The bladder was punctured with a 19G butterfly needle and emptied. It was expected 30 minutes for stabilization of the detrusor muscle. The needle was then connected to a saline infusion pump (4 ml / h). The bladder pressure record (Power Lab v. 5.0 System - AD Instruments, Australia) occurred for 40 minutes^16^. After this period, the animals were sacrificed by hypovolemic shock caused by the section of the abdominal aorta.

###  Variables analyzed 

 DO was defined as an increase in non-voiding contractions and micturition frequency, according to the following definitions:

- Non-voiding contractions (NVC): number of detrusor contractions not followed by voiding, prior to the first voiding. Non-voiding contraction was any rise in intravesical pressure above minimum (4 mmHg) that did not result in urine leakage;
- Threshold pressure (TP): detrusor pressure immediately before first micturition;
- Baseline pressure (BP): detrusor pressure immediately before micturition-related contractions;
- Frequency of micturition (FM): number of micturitions divided by time. During the 40-min evaluation, all voidings were registered. Detrusor contractions resulting in drops of urine were counted as voidings;
- Threshold volume (TV): infused volume immediately before first micturition.

###  Statistical analysis 

 The normality of the distribution of quantitative and continuous variables was confirmed in all cases with the Kolmogorov-Smirnov test. The data were submitted to descriptive statistics and mean values and standard deviations were calculated. Variance analysis (ANOVA) was used to compare the groups with regard to specific variables. Paired comparisons were performed with Tukeyʼs multiple comparison test. The level of statistical significance was set at 5% (p<0.05). All analyses and graphs were made with the software Graph Pad Prism v.5.00 for Windows (GraphPad Software, San Diego, California, USA, 2007).

## Results

 The cystometric findings are summarized in Table 1.

**Table 1:** Cystometric parameters.

Parameter	Non-voiding contractions NVC (n)	Threshold pressure TP (mmHg)	Baseline pressure BP (mmHg)	Freq. of micturition FM (cycles/min)	Threshold volume TV (mL)
Group 1 Control	1.50 ± 0.55	56.62 ± 23.31	9.90 ± 6.38	0.69 ± 0.56	0.27 ± 0.14
Group 2 L-NAME	4.33 ± 2.58^a^	36.02 ± 13.91	17.45 ± 2.62	2.18 ± 0.68^b^	0.34 ± 0.26
Group 3 L-NAME + tadalafil	2.00 ± 1.10	32.61 ± 8.65^a^	11.21 ± 5.67	0.97 ± 0.71^d^	0.55 ± 0.44
Group 4 L-NAME + BRL37344	1.50 ± 1.52^c^	28.95 ± 9.51^a^	14.86 ± 4.17	0.92 ± 0.38^d^	0.24 ± 0.04
Group 5 L-NAME + Tadalafil + BRL37344	2.00 ± 1.26	25.62 ± 4.92^b^	14.84 ± 3.65	1.05 ± 0.44^c^	0.28 ± 0.13
P-value	0.0218	0.0051	0.05	0.0012	0.1978

**Figure 1:**
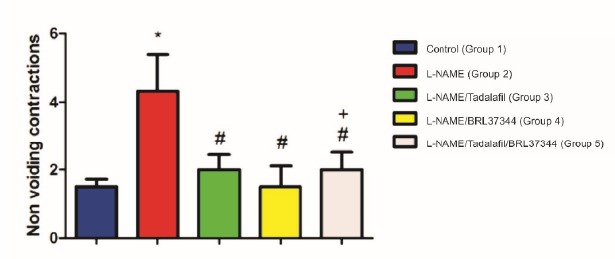
Number of non-voiding contractions. *Significant difference in relation to Group 1, 3, 4 and 5. # + significant difference in relation to Group 2. # Group 5 was statistically similar to Groups 3 and 4.

 NVC was significantly higher in Group 2 than in Group 1, 3, 4 and 5. The combination of tadalafil and BRL 37344 produced no measurable additive effect on detrusor smooth muscle relaxation. Groups 3 and 4 were statistically similar to Group 5 (p>0.05) (Fig. 1). TP was significantly lower in Group 3, 4 and Group 5 than in Group 1 (p<0.005). BP was lower in Group 3, 4 and 5 than in Group 2 (p=0.05). TV was similar in the five groups (p=0.19) (Table 1).

**Figure 2:**
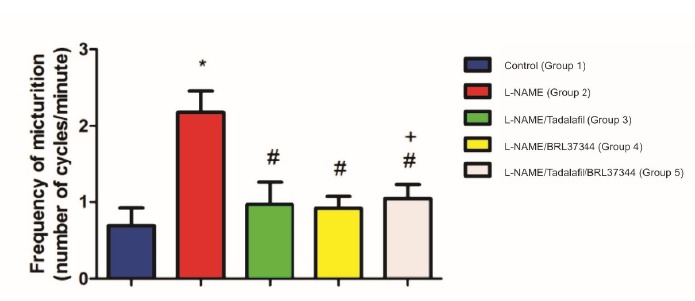
Frequency of micturition. *Significant difference in relation to Group 1. # significant difference in relation to Group 2. + # produced no measurable additive effect on detrusor smooth muscle relaxation.

 The FM increased with L-NAME (Group 2) and decreased with tadalafil and BRL 37344 (Groups 3, 4 and 5). The combination of tadalafil and BRL 37344 produced no measurable additive effect on detrusor smooth muscle relaxation. FM was significantly higher in Group 2 than in Group 1 (p<0.01), and significantly lower in Group 3, Group 4 and Group 5 than in Group 2 (p<0.05) (Fig. 2). 

**Figure 3:**
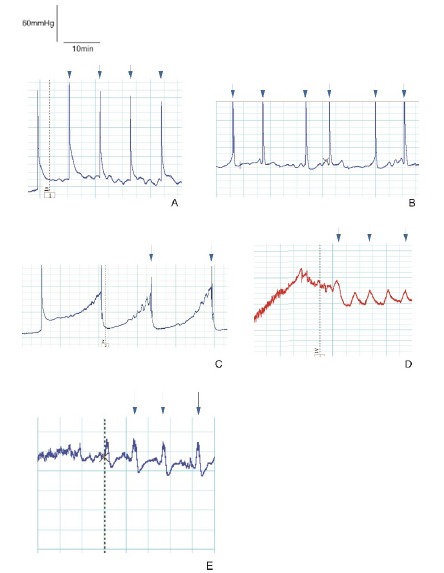
Representative cystometrograms. **A**=Group 1 (control); **B**=Group 2 (L-NAME); **C**=Group 3 (L-NAME + tadalafil); **D**=Group 4 (L-NAME + BRL 37344); **E**=Group 5 (L-NAME + tadalafil + BRL 37344). The arrows indicate micturition peaks.

Figure 3 shows the representative cystometrograms of all groups.

## Discussion 

 In vivo animal cystometry represents an accepted methodology for the study of lower urinary tract physiology. Mice are technically more difficult to use, but the same approach as in rats can be used. Suprapubic voiding cystometry using a simple and reliable urine collection method under urethane anesthesia is feasible in mice, permitting the integration of voided volumes with pressure and time data. The inclusion of volume and flow data enhances the usefulness of the mouse model for in vivo assessment of DO. Available disease models in rodents have limited translational value, but despite many limitations, rodent cystometry may give important information on bladder physiology and pharmacology^17^.

 Consistent methods for performing lower urinary tract function testing in mice are required to compare results among studies with confidence. Differences in results of lower urinary function testing vary among strains of mice and between males and females of the same strain^18^. Interpretation of cystometric data is complicated by the fact that it is often performed in different ways by different laboratories. Methodological variables include varying concentrations of urethane for anestesia^19^, length of rest period following surgery, infusion for different times prior to beginning measurement, tubing diameter, and methods of insertion/suturing of the catheter. These variations in methodology and analysis create problems when comparing results gathered from different laboratories^18^. Micturitions in rodents and humans differ significantly and this must be considered when cystometry is used to interpret voiding in rodent models. Cystometry in humans requires active participation of the investigated patient (subject), and this can for obvious reasons not be achieved in the animals. Cystometric parameters in rodents are often poorly defined and do not correspond to those used in humans^17^.

 In an in vivo experimental study, DO caused by administration with L-NAME was confirmed by cystometry, systemic reduction of NO causes DO and acute infusion of PDE-5 sildenafil reduces the number of micturition cycles in chronic NO-deficient rats^20^. Regadas *et al*.^21^, evaluated the urodynamic effects in the treatment of patients with lower urinary tract symptoms (LUTS). Tamsulosin (0.4 mg) with or without tadalafil (5 mg) were given to patients for 30 days. It has been observed that the tamsulosin/tadalafil combination reduces the detrusor pressure at maximum flow without changing the maximum flow rate during micturition and significantly improves LUTS compared with the isolated use of tamsulosin.

 One study has experimented, observed the effect of combination of tadalafil with tamsulosin on the lower urinary tract of rats with bladder outlet obstruction induced by chronic nitric oxide deficiency with L-NAME. After 30 days the animals were submitted to urodynamic study. Tadalafil did not cause impairment in detrusor muscle and seems to have an addictive effect to tamsulosin because the combination decreased non voiding contractions as well the number of micturition cycles^22^. In the corpus cavernosum of the penis, PDE-5 inhibition enhances relaxation of smooth muscle induced by NO and cGMP, and thereby stimulates penile erection^23^.

 The β3-AR agonists are the most notable alternative class of agents to antimuscarinics in the pharmacological treatment of overactive bladder. The β3-AR agonists act to facilitate bladder storage function probably through at least two mechanisms: first, direct inhibition of the detrusor, and second, inhibition of bladder afferent neurotransduction^24^.

 Fujimura *et al.* ^25^, induced the DO with ibutronic acid injection and tested the oral administration of β-adrenergic agonist FK-175 at a dose of 10mg / kg. it was observed a significantly increased bladder capacity, with no increase in urination pressure or pressure threshold. Likewise, cystometry in rats after intravenous administration of CL316243, another β3 adrenergic agonist, increased bladder capacity without increasing residual volume^26^. In the present study, we defined other parameters to be studied: FM and NVC.

 Activation of β3-AR by Mirabegron relaxes DSM, improving bladder compliance and increasing bladder capacity. There is no change in urination pressure and post-urination residue. It acts on the spontaneous contractility activity which occurs during bladder filling, whereas contraction of urination that depends on the parasympathetic discharge of the sacral medulla is not affected. The most common adverse effects recorded are dry mouth (placebo level) and gastrointestinal disturbances, rated as mild to moderate^27^. Mirabegron leads to an improvement in episodes of incontinence and frequency of urination similar to that observed in patients with or without prior anti-muscarinic therapy for OB^28^.

 While L-NAME and tadalafil were administered chronically, BRL 37344 was administered acutely. The decision was based on an experimental study in which a single intraperitoneal administration of BRL 37344 (5 mg/kg) decreased FM by 40-70% in rats with DO induced by ovariectomy^15^. Experimental studies with rats have shown that the β3 adrenergic agonist CL316243 may directly inhibit DSM contractility, experimental hyper reflex and detrusor instability, and be useful for urge urinary incontinence^6^. CL316243 can also suppress DO without increasing the volume of post-urination residue and cardiovascular adverse effects^29^.

 A selective β-agonist and a selective phosphodiesterase inhibitor appear to form an excellent combination for relaxation of DSM. The use of drug combinations appears to be a trend in order to treat LUTS patients more broadly^30^. No other study has tested the combination of BRL 37344 and tadalafil in models of DO in vivo.

 An in vitro study evaluated the effect of the combination of BRL 37344 and tadalafil or rolipram (phosphodiesterase type 4 inhibitor) in an experimental model of DO. The experiments were carried out in two phases using bladder strips of mice. In the first phase, on the top of 40 mM potassium-induced contraction, strips isolated from control mice were exposed increasing concentrations of each study drug. In another series of experiments, prior to contraction, strips were incubated with either tadalafil or rolipram, followed by the addition of increasing concentrations of BRL 37344. In the second phase, the same protocols were performed with animals previously treated with L-NAME for 30 days. In phase one, preincubation with tadalafil enhanced relaxation response to BRL 37344 at two concentrations (100 nM e 10 µM). Pretreatment with rolipram had no effect on BRL 37344-induced relaxation. In L-NAME treated mice, rolipram induced more relaxation than the other drugs, enhancing relaxation response to BRL 37344 at almost all concentrations, but no synergistic effect with tadalafil was observed. The relaxant effect of BRL 37344 was enhanced by rolipram but not by tadalafil, suggesting that PDE-4 inhibition, especially when associated with β3-AR, could represent a potential treatment for DO^31^. Thus, other studies using the rolipram and BRL 37344 combination may contribute to the treatment of DO.

 Our study was limited by the small sample size (in compliance with ethical guidelines), a group of animals should receive standard gold treatment for comparison with the proposed new treatment options and by the administration of maximum doses of β3-AR agonists. The principles of 3Rs (Reduction, Refinement and Replacement) are based on finding alternatives to reduce the number of animals in research^32^. Results may have been different had the sample been larger. Also, by administering maximum doses of each drug, the ceiling effect may have been reached individually, making it impossible to detect an additive effect. Studies testing drugs at multiple concentrations are necessary to clarify this issue. β3-ARs in the urothelium may contribute to the regulation of bladder function, but a molecular biological function has not been demonstrated. Future studies should measure NO, cAMP and cGMP and relate findings with data obtained from urodynamics.

## Conclusion 

 The combination of BRL 37344 and tadalafil produced no measurable additive effect on reduction of OB symptoms after induction of DO.
